# Supplementary material for: Plant-Based Diet, Cholesterol, and Risk of Gallstone Disease: A Prospective Study
Source: Nutrients. 2019 Feb 4;11(2):335. doi: 10.3390/nu11020335 (PMC6412457; doi:10.3390/nu11020335)
Supplement: Supplementary file 1 [file nutrients-11-00335-s001.pdf]

Supplemental Materials

Table S1. Sensitivity test by eliminating symptomatic GSD cases diagnosed within one year of enrollment.

		**All**	**Male**	**Female**
Vegetarian	Cases / Person-years	75 / 29284	33 / 11960	42 / 17324
	HR (95% CI)	0.62 (0.34, 1.13)	1.26 (0.51, 3.14)	0.42 (0.19, 0.93)

Adjusted for age, sex, education, smoking status, alcohol, sport, diabetes, chronic kidney disease, BMI, lipid-lowering medications, menopause (for female)

Abbreviation: HR, hazard ration; CI, confidence interval.
